# Descriptive Study of the Clinical and Etiological Profiles of Palmoplantar Dermatoses in Patients Attending a Tertiary Care Hospital in Southern India

**DOI:** 10.7759/cureus.20268

**Published:** 2021-12-08

**Authors:** Vijay Sekhar P, K Narasimha Prasad, D Subhash Reddy, Praveen Kumar Boppani

**Affiliations:** 1 Dermatology, Government Medical College, Mahabubnagar, IND; 2 Dermatology, Government Medical College, Nalgonda, IND; 3 Dermatology, Mallareddy Medical College for Women, Hyderabad, IND; 4 Dermatology, Central Hospital, South Central Railway, Hyderabad, IND

**Keywords:** palmoplantar dermatoses, hand eczema, palmoplantar keratoderma, tinea manuum, fungal infections, psoriasis

## Abstract

Introduction

Palmoplantar dermatoses are skin diseases affecting specifically palms and soles is a common clinical entity encountered by dermatologists. This may cause discomfort and embarrassment to the patients because its location interferes significantly with normal day-to-day activities. Palmoplantar dermatosis affects a heterogeneous population, ranging from bare-handed workers in the slaughterhouse to medical personnel wearing gloves in the operating room. The present study endeavors to classify palmoplantar dermatoses based on their morphology and the clinical distribution pattern.

Materials and methods

The present study is a descriptive cross-sectional study conducted for six months at Osmania General Hospital, India. A total number of 80 patients having palmoplantar dermatoses who met the study inclusion criteria were included. After taking proper informed consent, data were collected from patients on a fixed proforma sheet.

Results

The study comprised 44 (55%) male and 36 (45%) female patients. The mean duration of palmoplantar dermatoses was 2.92 years. Itching and painful fissures are the most common symptoms patients complained of and psoriasis is the most common clinical type seen in 32 (40%) patients followed by eczemas in 30 (37.5%) patients and fungal infections in 10 (12.5%) patients.

Conclusion

The term palmoplantar dermatoses includes a heterogeneous group of skin disorders but no exact classification is present and classes differ based on the diseases to be kept in a group. Physicians should be well aware of the clinical features of these dermatoses and diagnosis is very important for early intervention and correct management, thereby helping improve patients' quality of life.

## Introduction

Palmoplantar dermatoses are skin diseases affecting specifically the palms and soles. It is a common clinical entity encountered by dermatologists. This may cause discomfort and embarrassment to patients because of its location and interferes significantly with normal daily activities. Palmoplantar dermatoses effects a heterogeneous population, ranging from bare-handed workers in slaughterhouses to medical personnel wearing gloves in the operating room. Detecting the cause requires good history-taking and patient orientation. The characteristic diagnostic features of clinical conditions, such as psoriasis, dermatophytosis, and eczemas, which are seen elsewhere on the body are usually masked or modified on palms and soles [1]. Hyperkeratosis is the usual morphological pattern seen in these areas, with papulovesicular eruptions coming next in order of frequency [2]. This makes accurate clinical diagnosis “a chance" possibility. Added to this, secondary changes that occur due to constant contact with various substances make the clinical picture confused. Confirming the diagnosis in a given case is often difficult and time-consuming and a battery of investigations are to be performed to come to a reasonable conclusion. The present study endeavors to classify palmoplantar dermatoses based on its morphology and to correlate the clinical type.

## Materials and methods

The present study was a prospective observational study carried out from July 2017 to December 2017 in the Department of Dermatology, Venerology, and Leprosy at a tertiary care hospital in South India. A total of 80 cases were enrolled for the six-month duration study, and the study was reviewed and approved by the institutional ethical committee of Osmania Medical college with IRB approval number 17104001012R. A sample size of 80 was selected, and this was calculated by using the formula 4pq/d2 where p=prevalence, q=100-p, and d=absolute error. All patients with palmoplantar dermatoses irrespective of lesions elsewhere on the body, of any age group and sex, and who were willing to participate in the study were included. Patients who were already on treatment and not willing to participate were excluded from the study. Informed consent was taken from all the cases before data collection.

After obtaining informed written consent in the patient's own language, complete history was obtained and a thorough clinical examination was done according to the case sheet proforma which is specially prepared for the study so as to arrive at a provisional diagnosis in each case. Laboratory investigations, such as complete blood picture, random blood sugar, potassium hydroxide (KOH) mount, patch testing, and histopathological examination, were done wherever required. Skin scrapings were taken from the involved sites of palms and soles from all the patients included in the study. Scrapings were collected from at least two sites to detect fungal elements, preferably one from the palm and the other from the sole if both palms and both soles are involved. The KOH mount technique - a drop of 10% potassium hydroxide is carefully placed on the edge of the coverslip and allowed to flow evenly beneath the coverslip by surface tension [2]. The slide is blotted dry and observed for fungal elements, which appear as septate and branching hyphae, under the microscope. The cases reported for positive fungal elements were investigated for identifying the causative fungus by performing culture studies by inoculation into Sabouraud's dextrose agar medium with added chloramphenicol 0.05 g/L and cycloheximide 0.4 g/L.

All the findings were tabulated and compared with each other and charts were prepared to arrive at the results. Descriptive statistics, such as frequency and mean, with standard deviation (SD), were calculated to present the results. Categorical variables were used to express the numbers and percentages.

## Results

As shown in Table 1, among the 80 cases studied, the mean age was 25.63 years and the standard deviation was 6.53. The 11-20-year age group is the largest (37.5%) and next in order of frequency is the 31-40-year age group (21.25%). More than half of the patients are in the 10-30-year age group. The study comprised 44 (55%) males and 36 (45%) females, and the male: female ratio was 1.2. Itching was the most common complaint of 64 patients (80%) and next in frequency was painful fissures (77.5%). Hyperhidrosis was complained of by 22 patients (27.5%) (Table 2).

**Table 1:** Age and gender distribution

Variables	No.of Patients (%)	
Age Group	0-10 years	9(11.25%)
	11-20 years	30(37.5%)
	21-30 years	11(13.75%)
	31-40 years	17(21.25%)
	41-50 years	6(7.5%)
	51-60 years	3(3.75%)
	61-70 years	4(5%)
	Mean age ± SD	25.63 ± 6.53
Gender	Male	44(55%)
	Female	36(45%)

**Table 2:** Presenting symptoms

S.No.	Symptom	Total No. of Patients Who Complained
1	Itching	64(80%)
2	Painful fissures	62(77.5%)
3	Hyperhidrosis	22(27.5%)
4	Soreness	22(27.5%)
5	Discharge seropurulent/Serous	18(22.5%)
6	Bad odor	6(7.5%)

Itching was complained of in 26 of 32 patients clinically diagnosed with psoriasis (81.25%), 25 patients out of a total of 30 patients with eczemas complained of itching (83.3%), eight of 10 patients with fungal infections complained of itching (80%), and three of six patients with hereditary keratodermas complained of itching (50%) (Table 3).

**Table 3:** Number of patients who complained of itching in different clinical types

Disease	Total No. of patients	No. of patients who complained of itching	No. of patients in whom itching was absent	Percentage of patients who complained of itching
Psoriasis	32	26	6	81.25%
Eczemas	30	25	5	83.3%
Fungal Infections	10	8	2	80%
Keratodermas	6	3	3	50%
Others	2	-	2	-

The mean duration of palmoplantar dermatoses was 2.92 years. The duration ranged from a minimum of less than one month to a maximum of 25 years in one case. More than half of the patients had dermatoses for six months to two years. Generalized dryness of skin was present in 22 patients and 10 patients gave a personal or family history of atopy. Hypertension was found in nine patients and diabetes mellitus in four patients.

Significant history of contact with and aggravation of the disorder by the use of detergents/sanitizer was found in 15 patients with hand involvement (24.19%) (significant means aggravation after particular use of a contactant and remission after stoppage of use of the same). A significant history of contact with vegetable juices and crushed vegetables was found in 14 patients (22.58%) (Table 4). Aggravation of dermatitis by the use of footwear was found in five patients (7.14%).

**Table 4:** Number of patients with a significant history of contact with irritants/sensitizers

S.No.	History	Total No. of Patients With involvement of the Hands	Total No. of Patients With involvement of the Feet
1	Significant history of contact with water	16 (25.80%)	1 (1.42%)
2	Significant history of contact with and aggravation by use of detergents/sanitizer	15 (24.19%)	1 (1.42%)
3	Significant history of contact with vegetable juices and crushed vegetables (kitchen dermatitis)	14 (22.58%)	-
4	Significant history of contact with irritants and solvents	2 (3.22%)	1 (1.42%)
5	History of aggravation of symptoms with footwear use	-	5 (7.14%)

Forty-six patients included in the study showed involvement of palms and soles (Table 5). Eighteen patients showed involvement of only soles, 10 showed only palmar involvement, 13 patients included the involvement of other areas of the body such as extensor surfaces of extremities and the face in one case. Similar morphological types of dermatitis, as those affecting palms and soles, are seen in other areas.

**Table 5:** Showing distribution of palmoplantar dermatosis

			Involvement	
S.No.	Distribution	Unilateral		Bilateral
1	Only palms	4		6
2	Only soles	-		18
3	Both palms/both soles	-		46
4	Both soles/one palm	-		6
5	Involvement of other areas along with palms and soles		13	

**Figure 1:**
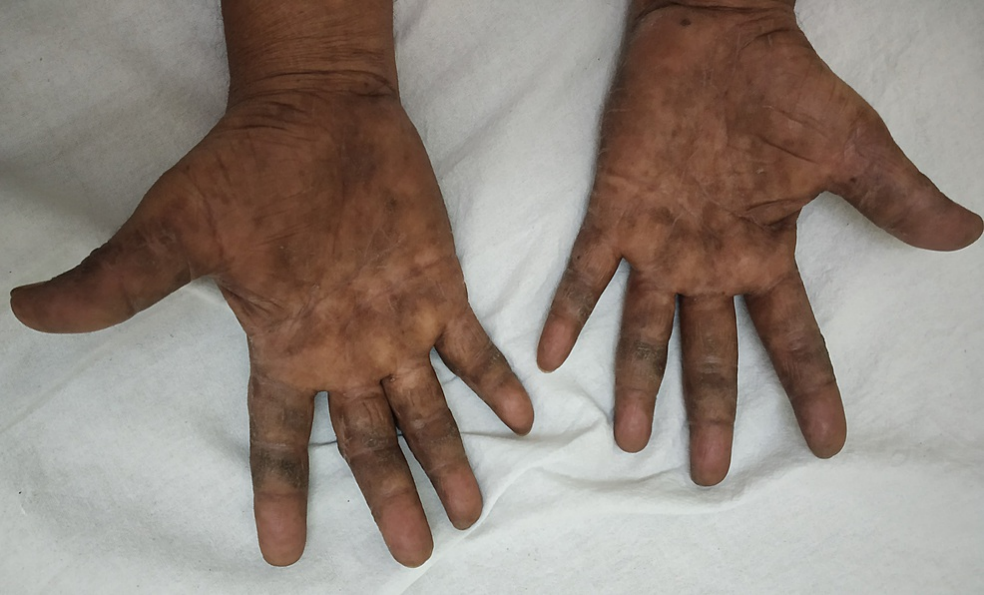
Allergic contact dermatitis

**Figure 2:**
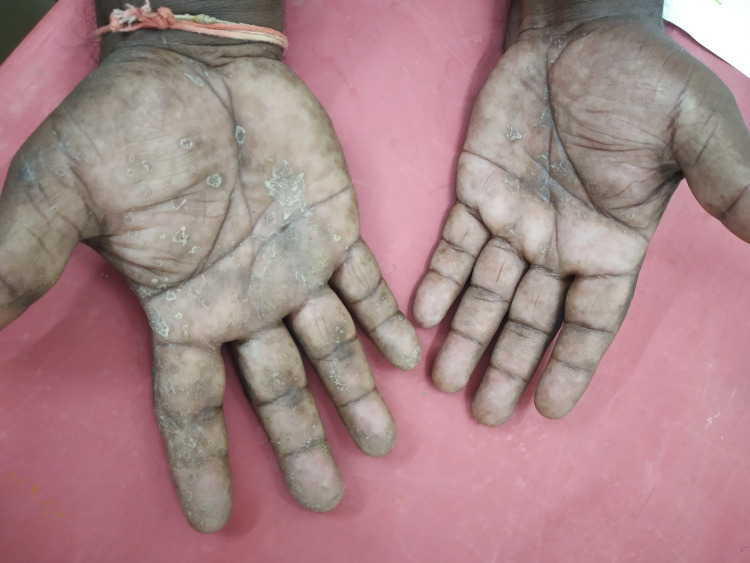
Tinea manuum

Psoriasis was the commonest disorder diagnosed clinically on the basis of morphology in 32 of 80 patients followed by eczemas (30) and fungal infections (10) (Figures 1-2). Six patients were diagnosed as keratodermas and one each of discoid lupus erythematosus and erythema multiforme (Table 6).

**Table 6:** Showing provisional diagnoses

S.No.	Provisional diagnosis	No. of Patients	Total
1	Psoriasis	32	32
2	Fungal Infections		10
	- Tinea manuum	4	
	- Tinea pedis	5	
	- Tinea manuum/pedis	1	
3	Eczemas		30
	- Contact Allergic Dermatitis	14	
	- Pompholyx	7	
	- Contact Irritant Dermatitis	5	
	- Atopic	1	
	- Juvenile Plantar Dermatosis	3	
4	Keratodermas		6
	- Hereditary (Diffuse-2 and Punctate-2)	4	
	- Associated with Ichthyosis vulgaris	2	
5	Others		2
	- Erythema multiforme	1	
	- Discoid lupus erythematosus	1	

The involvement of the palms was patchy in 43 patients (69.35%) and diffuse in 12 patients (19.35%), six patients had only fingertip involvement (9.69%), and one patient showed hyperkeratotic plaques limited to creases (1.6%). As shown in Table 7, in the morphological types, hyperkeratotic plaques with fissures were present in 42 cases (67.74%), dry discoid plaques in eight patients (12.9%), and papulovesicular or pustular lesions in 11 patients (17.74%). One patient showed hyperpigmented macules in the center of both palms (1.6%).

**Table 7:** Showing correlation of distribution with Morphological type on palms

Morphology Distribution	Papulovesicular or Pustular	Dry discoid	Hyperkeratotic with fissures	Pigmented	Total (%)
Fingertip	1	-	5	-	6 (9.69%)
Diffuse or generalized	2	3	7	-	12(19.35%)
Patchy	8	5	29	1	43(69.35%)
Creases	-	-	1	-	1
Total (Percentage)	11 (17.74%)	8 (12.9%)	42 (67.74%)	1 (1.6%)	62

**Figure 3:**
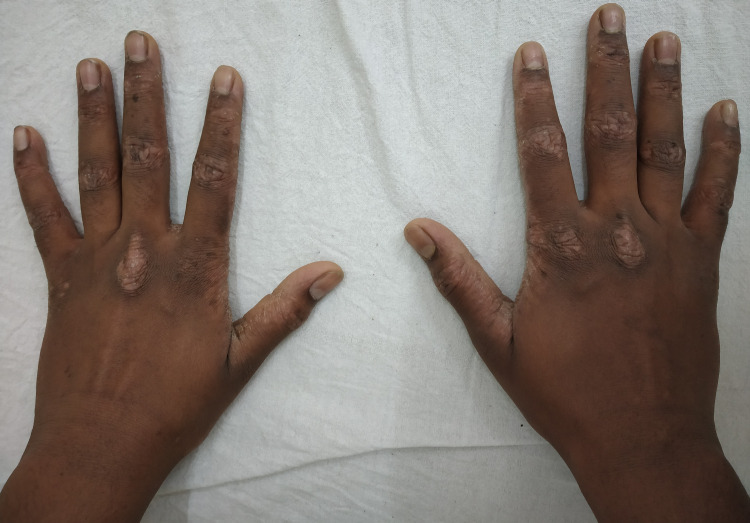
Palmar psoriasis showing extension onto the dorsum of the hands

Twenty-five patients with palmar dermatoses showed extension on to the dorsum of the hands (40.98%) (Figure 3), while seven patients of vesicular dermatitis (63.63%), seven patients of dry discoid plaque type (87.5%), and 11 patients of hyperkeratotic plaque type (26.19%) showed dorsal extension. All fingertip eczema patients showed extension onto the dorsum (100%) while five patients of diffuse-type (41.6%) and 14 of patchy-type involvement showed dorsal extension (32.5%).

Involvement of soles was predominantly peripheral or marginal in 23 patients (32.85%), 15 patients (21.42%) showed mostly involvement of the instep region, eight patients (11.4%) showed the involvement of weight-bearing areas, diffuse involvement was seen in 11 patients (15.7%), and patchy involvement in five patients (7.14%). In the morphological types, hyperkeratotic plaques with fissuring were noted in 55 patients (78.57%), dry discoid in nine (12.85%), and papulovesicular or pustular lesions in six patients (8.57%) (Table 8).

**Table 8:** Correlation of distribution with morphological type on the soles

S.No.	Morphology ---> Distribution	Papulovesicular or pustular	Dry discoid	Hyperkeratotic with fissures	Total %
1	Marginal or peripheral	2	3	18	23 (32.85%)
2	Instep	1	2	12	15 (21.42%)
3	Diffuse	2	2	7	11 (15.7%)
4	Weightbearing	-	-	8	8 (11.4%)
5	Patchy	-	1	4	5 (7.14%)
6	Forefoot	1	-	3	4 (5.71%)
7	Peri digital	-	1	1	2 (2.85%)
8	Hindfoot	-	-	2	2 (2.85%)
	Total (Percentage)	6 (8.57%)	9 (12.85%)	55 (78.57%)	70

## Discussion

Many inflammatory and non-inflammatory dermatoses assume hyperkeratotic forms, especially on the palms and soles. This tendency is most evident in middle-aged patients. In some cases, the lesions, although modified, can readily be recognized but in others, only careful inspection of other areas will allow the clinical diagnosis to be established. Investigations such as skin biopsy from the lesions and bacteriological and mycological tests can help greatly in the confirmation of the diagnosis that has been attempted in the present study.

Psoriasis of palms and soles is a disease of middle-aged adults. Nair PA et al. found that the maximum number of patients with psoriasis of palms and soles were between 21 and 40 years [3]. Lakshmi C and Srinivas CR found the mean age of hyperkeratotic eczema to be 46 years [4]. In an Indian study by Khandpur et al., the average age group for psoriasis of palms and soles was 21-50 years [5]. In our study, the maximum number of cases seen were in the 10-30-year age group, which is not at variance with the above study as all the diseases that can cause palmoplantar dermatoses are included in the study.

In the present study, the ratio of affection of palmoplantar dermatoses in males and females is about 5:4 in favor of males. A similar predominance of males over females was observed in other studies like Khandpur et al. [5] and Rathoriya SG et al. [6]. Most cases in the present study had a duration of dermatoses ranging from one to two years with a mean of 2.92 years. A similar trend was observed in the study by Khandpur et al. [5].

Itching was the predominant symptom complained of by 64 out of 80 patients (80%), which was similar to other studies done by Kumar B et al., Venkatesan et al., and Gutte et al. [7-9]. Although it is reported that itching is very variable, ranging from complete absence to severe pruritus in a minority of patients, in the present study, 81.25% of patients clinically diagnosed with psoriasis complained of itching. Eighty percent of patients in whom fungal infection of palms and soles was clinically diagnosed also complained of itching. The other chief complaint was painful fissures, which was elicited in 62 out of 80 patients (77.5%) in our study. Hyperhidrosis and soreness were complained of by 22 patients in the study.

A significant history of contact with water, detergents/sanitizers, which lead to initiation and/or aggravation of palmoplantar dermatoses, was elicited in 17 out of 80 patients (21.25%) under study. Significant aggravation of palmar dermatoses on contact with vegetable juices and crushed vegetables was elicited in 14 out of 52 patients (26.92%), indicating the importance of kitchen dermatoses. History of aggravation of plantar dermatoses by a recent change in footwear was complained of by five out of 70 cases (7.14%) of plantar dermatoses.

Generalized dryness of skin was noted in 22 of 80 patients in the present study. Personal or family history of atopy could be elicited in 8% of patients in the present study. No significant association of any disease was observed, even though four patients had associated diabetes mellitus and nine patients had hypertension.

Bilateral lesions were seen in 76 out of 80 cases. In psoriasis of the palms and soles and hyperkeratotic eczema, the lesions are known to be bilateral. In the present study, a similar trend is observed as more than 75% of cases belonging to the above groups. Involvement of both palms and soles bilaterally and symmetrically was seen in 46 of 80 patients (57.5%) under study.

The clinical diagnosis of psoriasis was made in 32 out of 80 patients (40%). Various types of eczemas in 30 patients (37.5%), fungal infections - tinea manuum, tinea pedis, or both - in 10 patients (12.5%) and keratodermas - hereditary or with associated ichthyosis vulgaris - in six patients (7.5%). A study done by RathoriyaSG et al. showed palmoplantar pustulosis with 23.2%, warts with 11.4%, pompholyx with 10.1%, palmoplantar keratoderma with 8.9%, and contact dermatitis with 8.0% cases [6], while in a study by Kodali et al., 52% cases were diagnosed as psoriasis, 31% as eczema, 2% as fungal infection and 1% as warts [10].

When the morphological type was correlated with the distribution of dermatoses, the following observations were made. On the palms, patchy hyperkeratotic eczema was the most common, seen in 29 out of 62 cases (46.77%). The generalized hyperkeratotic type was seen in seven out of 62 cases (11.29%), being the second most common pattern observed, whereas on the soles marginal or peripheral pattern with hyperkeratosis and fissuring is the commonest pattern, seen in 18 out of 70 cases (25.71%). The second most common type was instep dermatoses with hyperkeratosis and fissuring (17.14%). Patchy dermatoses were not very common unlike on palms occurring in five out of 70 cases (7.14%).

Hyperkeratotic dermatoses with fissuring was the most common morphological type seen in 55 out of 70 cases (78.57%) of plantar dermatoses and in 42 of 62 cases (67.74%) of palmar dermatoses. Extension of dermatitis onto the dorsum of hands and the involvement of finger webs is seen in cases of contact dermatitis - either irritant or allergic. Dorsal extension of palmoplantar dermatoses was seen in a good number of cases in the present study. A total of 25 patients with palmar dermatoses and 33 patients with plantar dermatoses have shown extension of dermatoses onto the dorsum. The morphological pattern remained the same on an extension to the dorsum on both palms and soles. In most cases, the involvement was marginal or patchy. In other studies, webspace involvement of palmoplantar psoriasis was seen in 28% by Khandpur et al. [5] and 8% by Kumar et al. [7]. A hyperkeratotic morphological pattern was the most common to show extension onto the dorsum in both palms and soles seen in 11 of 25 patients (44%) with palmar dermatoses and 21 of 33 patients (63.63%) with plantar dermatoses. When palmar and plantar dermatoses were compared with each other on the basis of morphology, the following observations were made. Out of a total of 80 cases under study, both palms and soles were involved simultaneously in 52 cases, whereas only palms were involved in 10 cases and only soles were involved in 18 cases. In the 52 cases where palmoplantar involvement was seen, the following observations were made: 35 patients (83.33%) showed a hyperkeratotic morphological pattern on both palms and soles. Three patients (33.33%) showed a dry discoid pattern and six patients (100%) showed a papulovesicular or pustular pattern on both palms and soles. In other words, 44 of 52 cases (84.6%) have shown an identical morphological pattern of dermatoses on both palms and soles. Dissimilarity in morphological patterns was observed in the remaining eight of 52 cases.

## Conclusions

The term palmoplantar dermatoses includes a heterogeneous group of skin disorders, but no exact classification is present and classes differ based on the diseases to be kept under the group. Itching is the most common symptom seen over the palms and soles, which can be secondary to other diseases like psoriasis, eczema, and fungal infections. The highest incidence of palmoplantar dermatoses was seen in the below 30 years age group, which may be related to more exposure to injuries, contact with allergens, and occupational exposure in this age group.

 It is very important to identify the signs/symptoms, causative agent, and the clinical and morphological features of palmoplantar dermatoses affecting the palms and soles, as early diagnosis is very important for early intervention and correct management, thereby helping in improvement in patients' quality of life.
